# Tobacco use prevalence, knowledge, and attitudes among newly diagnosed tuberculosis patients in Penang State and Wilayah Persekutuan Kuala Lumpur, Malaysia

**DOI:** 10.1186/1617-9625-8-3

**Published:** 2010-01-12

**Authors:** Ahmed Awaisu, Mohamad Haniki Nik Mohamed, Noorizan Abd Aziz, Syed Azhar Syed Sulaiman, Noorliza Mohamad Noordin, Abdul Razak Muttalif, Aziah Ahmad Mahayiddin

**Affiliations:** 1 Department of Clinical Pharmacy, School of Pharmaceutical Sciences, Universiti Sains Malaysia, 11800 Penang, Malaysia; 2 Department of Pharmacy Practice, Kulliyyah of Pharmacy, International Islamic University Malaysia, 25200 Kuantan, Pahang DM, Malaysia; 3 Department of Health Economics & Finance, Institute for Health Management, NIH, Ministry of Health, 59000 Kuala Lumpur, Malaysia; 4 Department of Respiratory Medicine, Penang Hospital, Jalan Residensi, 10990 Penang, Malaysia; 5 Institut Perubatan Respiratori, 53000 Wilayah Persekutuan Kuala Lumpur, Malaysia

## Abstract

**Background:**

There is sufficient evidence to conclude that tobacco smoking is strongly linked to tuberculosis (TB) and a large proportion of TB patients may be active smokers. In addition, a previous analysis has suggested that a considerable proportion of the global burden of TB may be attributable to smoking. However, there is paucity of information on the prevalence of tobacco smoking among TB patients in Malaysia. Moreover, the tobacco-related knowledge, attitudes, and behaviors of TB patients who are smokers have not been previously explored. This study aimed to document the prevalence of smoking among newly diagnosed TB patients and to learn about the tobacco use knowledge and attitudes of those who are smokers among this population.

**Methods:**

Data were generated on prevalence rates of smoking among newly diagnosed TB patients in the State of Penang from January 2008 to December 2008. The data were obtained based on a review of routinely collated data from the quarterly report on TB case registration. The study setting comprised of five healthcare facilities (TB clinics) located within Penang and Wilayah Persekutuan, Kuala Lumpur health districts in Malaysia, which were involved in a larger project, known as SCIDOTS Project. A 58-item questionnaire was used to assess the tobacco use knowledge, attitudes and behaviors of those TB patients who were smokers.

**Results:**

Smoking status was determinant in 817 of 943 new cases of TB from January to December 2008. Of this, it was estimated that the prevalence rates of current- and ex-smoking among the TB patients were 40.27% (329/817) and 13.95% (114/817), respectively. The prevalence of ever-smoking among patients with TB was estimated to be 54,220 per 100,000 population. Of 120 eligible participants for the SCIDOTS Project, 88 responded to the survey (73.3% response rate) and 80 surveys were analyzed (66.7% usable rate). The mean (± SD) total score of tobacco use knowledge items was 4.23 ± 2.66 (maximum possible score=11). More than half of the participants (51.3%) were moderately dependent to nicotine. A moderately large proportion of the respondents (41.2%) reported that they have ever attempted to quit smoking, while more than half (56.3%) have not. Less than half (47.5%) of the study participants had knowledge about the body system on which cigarette smoking has the greatest negative effect. The majority wrongly believed that smokeless tobacco can increase athletic performance (60%) and that it is a safe and harmless product (46.2%). An overwhelming proportion (>80%) of the patients believed that: smoking is a waste of money, tobacco use is very dangerous to health, and that smokers are more likely to die from heart disease when compared with non-smokers. The use of smokeless tobacco was moderately prevalent among the participants with 28.8% reporting ever snuffed, but the use of cigar and pipe was uncommon.

**Conclusion:**

Smoking prevalence rate is high among patients with TB in Malaysia. These patients generally had deficiencies in knowledge of tobacco use and its health dangers, but had positive attitudes against tobacco use. Efforts should be geared towards reducing tobacco use among this population due to its negative impact on TB treatment outcomes.

## Background

Tuberculosis (TB) and tobacco use are regarded as two colliding epidemics of public health importance [1]. Recent estimates have shown that the two formidable epidemics kill more than six million people worldwide annually [2,3]. Furthermore, in recent years, there has been a global explosion of interest on the association between TB and exposure to tobacco smoke. Studies have unequivocally documented consistent evidence that smoking is associated with an increased risk of TB. Considerable information exists on the risks of latent TB infection, active disease and mortality from TB due to tobacco smoking in many countries of the world [4-7]. In fact, these evidences are growing at an alarming rate. For instance, three recent systematic reviews and meta-analyses of observational studies have reaffirmed that tobacco smoking is an important risk factor for being infected with *Mycobacterium tuberculosis*, progression to clinical disease and dying from TB [4-6]. These reviews reported a pooled odds ratio (OR) of between 1.8 and 2.1 for latent TB infection; a relative risk (RR) of about 1.6 for TB infection; a pooled OR of 2.6 for TB disease and; a RR of 2.3-3.3 for TB disease for both current- and ever-smokers [4-6]. Tobacco smoking has in addition found to be significantly associated with treatment failure, default, and relapse after successful TB treatment [8-11].

In parallel, tobacco smoking has increased substantially over the past few decades, in developing countries where TB is co-prevalent with an estimated 930 million of the world's 1.1 billion smokers currently living in the low-income and middle-income countries [12-14]. Furthermore, a large proportion of TB patients may be active smokers or involuntarily exposed to other people's tobacco smoke [12,15-18]. A recent case-control study from China reported a proportion of cigarette smoking of 54.6% among TB patients, which was significantly higher than that in the control group (45.1%) with an adjusted OR of 1.93. With the overwhelming and accumulating evidence of association between TB and tobacco smoking, TB control programs might benefit from integrated interventions aimed at reducing tobacco, especially among those at high risk [1,4,19]. Therefore, patients diagnosed with TB who are smokers need to be aware of this association and the implications of continued smoking on short- and long-term TB treatment outcomes as well as future lung health. They also need to be well-educated about tobacco use and its health dangers in general as well as posses positive attitudes towards reducing tobacco smoking. Understanding the tobacco use literacy, attitudes and behaviors of newly diagnosed TB patients is of paramount importance in individualizing smoking cessation intervention via behavioral therapy and in designing effective educational intervention programs for the prevention of TB, treatment failure, recurrence after successful treatment and other poor outcomes.

Information on the prevalence rates of smoking among TB patients in general and specifically in Malaysia is scarce. Moreover, the tobacco-related knowledge, attitudes, and behaviors of TB patients who are smokers have not been previously investigated. This study aimed to determine the prevalence of smoking among newly diagnosed TB patients and to evaluate the tobacco use knowledge and attitude of those who are smokers among this population.

## Methods

### Prevalence of Smoking among Patients Newly Diagnosed with TB

In this study, we generated data on smoking rates among newly diagnosed TB patients for the State of Penang in north Malaysia from January through December 2008. The data were obtained based on a review of routinely collated data from the quarterly report on TB case registration from various basic management units. Clinicians working in government TB clinics in the State of Penang were requested to routinely document the smoking status of newly diagnosed TB patients using pre-determined standard definitions, modified from Centers for Disease Control (CDC). Current smoker was defined as a patient who has smoked at least 100 cigarettes in his or her lifetime and who still smokes daily or occasionally at the time of TB diagnosis or has recently stopped within the period of experiencing the current symptoms of respiratory illness. Ex-smoker was defined as a person who reported smoking at least 100 cigarettes in lifetime, but has stopped at least one month before experiencing the current symptoms of TB. Current and ex-smokers were considered as ever smokers (a person who has reported to have smoked at least 100 cigarettes in lifetime). Patients whose smoking status was not documented were excluded from the analysis. For the estimation of the smoking rates among patients with TB, the numerator was the number of patients who fulfilled the definition of self-reported smoking as above (ex- or current smoker), and the denominator was the total number of patients in the cohort whose self-reported smoking status was recorded during TB diagnosis.

### Tobacco Use Knowledge and Attitudes Survey among TB Patients who Smoke

#### Study Area

The study area comprised of five healthcare facilities (TB clinics): four centers located within Penang State and one center in Wilayah Persekutuan Kuala Lumpur health districts. The five clinics were involved in a larger project, known as SCIDOTS Project. The larger project was primarily intended to establish a value-added service (delivery of smoking cessation intervention by TB-DOTS providers) for patients with TB and to evaluate its direct impact on the outcomes of tobacco cessation and TB treatment.

#### Study Design and Population

This was a cross-sectional survey targeting all subjects planned to be involved in an interventional study. The study population included all the TB patients who were current smokers at the time of TB diagnosis and who were eligible and consented to be enrolled in the SCIDOTS Project. Both patients in the preparation stage of behavior change (intervention group) and those in the pre-contemplation/contemplation stage of behavior change (control group) were approached to participate in the survey on a voluntary basis.

#### Instrument

A 58-item questionnaire was used for the survey. The questionnaire comprised of four sections: socio-demographic and smoking-related information (12 items), knowledge of tobacco use (11 items), tobacco use attitudes (18 items), and practice of tobacco use (17 items). Multiple choice response format was used for the knowledge questions, with one correct answer for each question. In addition, the 18-item attitudes domain used a five-point rating scale that indicated degrees of agreement (strongly agree, agree, neutral, disagree, and strongly disagree).

The questionnaire was initially developed in English based on a study by Torabi et al. [20] and the Tobacco Attitude Scale developed by Meier [21]. A panel of experts in the field of public health and tobacco control reviewed the questionnaire for content and face validity. Changes were made to clarify any ambiguity and to ensure comprehension of the target population. Standard forward and backward translation procedures were used to develop a conceptually and culturally equivalent Malay version of the questionnaire. The translation committee comprised of the researchers and two independent professional translators (natives of Malay) who are fluent in both English and Malay. The final reconciled version of the questionnaire in Malay was produced and pilot tested among 20 smokers before use in the survey. The reliability of the knowledge and attitude domains of the survey instrument was tested using Cronbach alpha technique. The reliability coefficients of the two domains were 0.783 and 0.721, respectively.

#### Data Collection Procedures

The questionnaire was administered during the first visit to TB clinic upon fulfillment of the eligibility criteria for recruitment into the SCIDOTS Project and signing of an informed consent. Participants were approached to answer the questionnaire on voluntary basis. Participation in the survey was not a condition for enrollment in the main project, but only subjects enrolled in the project were required to participate in the survey. All survey participants were assured of anonymity and confidentiality. Approval for the conduct of the study was obtained from Medical Research Ethics Committee (MREC) of the Ministry of Health, Malaysia.

#### Data Analysis

Data were stored in Microsoft Excel and subsequently analyzed by using SPSS version 15.0 software package (SPSS Inc., Chicago, IL). Exploration of the data was performed prior to analysis to determine missing values and the distribution (normality) of the variables. Both descriptive and inferential statistics were applied wherever appropriate. Descriptive statistics were used to describe patients' demographic information, knowledge, attitudes and tobacco use behaviors. Knowledge on tobacco use was evaluated using total score for each participant, with one point for each correct answer and zero points for each wrong answer; the possible score in knowledge domain for each subject ranged from zero to 11. Student's *t*-test and ANOVA test were applied to determine demographic differences in knowledge among the respondents.

## Results

### Prevalence of Smoking among Newly Diagnosed TB Patients

**Figure 1:**
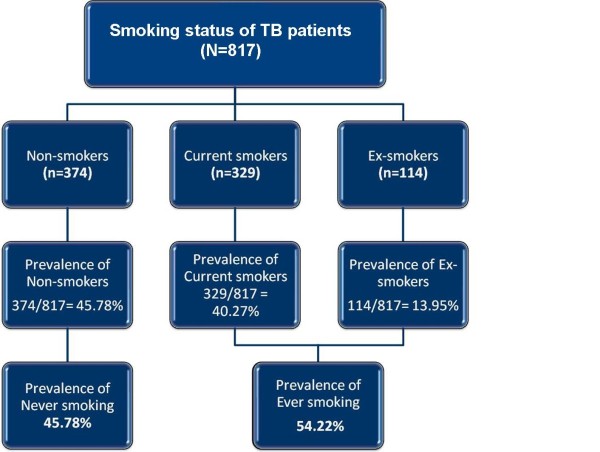
**Prevalence of Smoking among Patients with TB in Penang State, Malaysia (January - December 2008)**.

There were 943 patients diagnosed with TB from January to December 2008 in the State of Penang. Of this, the smoking status of 126 patients (13.36%) was not documented by the clinicians; hence these patients were excluded from the smoking prevalence estimations. The rates of current-smoking and ex-smoking among the TB patients were 40.27% (329/817) and 13.95% (114/817), respectively. The prevalence of ever-smoking among patients with TB was estimated to be 54,220 per 100,000 population. Figure 1 illustrates the smoking status and rates of the newly diagnosed TB patients in the Penang State.

### Tobacco Use Knowledge, Attitudes and Practices of TB Patients Who Smoke

#### Demographic and Smoking-related Characteristics

Of the 120 patients enrolled in the SCIDOTS Project, 88 (73.33%) agreed to participate in the tobacco use KAP survey. Of this, 80 surveys were included in the data analysis (usable rate of 66.67%). Table 1 provides the socio-demographic characteristics of the study respondents. Of the 80 participants, 56 (70.0%) were Malay, 18 (22.5%) were Chinese, and only four (5.0%) were Indian. The respondents were predominantly male (98.7%), which reflects the low smoking rate among female in Malaysia. Nearly 61.3% of the patients were 42 years or older at the time of recruitment into the study.

**Table 1:** Socio-demographic characteristics of the newly diagnosed TB patients who smoke (N = 80)

Characteristic	n (%)
	
***Gender***	
Male	79 (98.7%)
Female	1 (1.3%)
	
***Age***	
18-25 years	10 (12.5%)
26-33 years	12 (15.0%)
34-41 years	9 (11.3%)
42-49 years	14 (17.5%)
≥50 years	35 (43.8%)
	
***Race***	
Malay	56 (70.0%)
Chinese	18 (22.5%)
Indian	4 (5.0%)
Others	2 (2.5%)
	
***Occupation***	
Government	4 (5.0%)
Private	35 (43.8%)
Self-employed	29 (36.2%)
Others	12 (15.0%)
	
***Living environment***	
An urban area	35 (44.9%)
A suburban area	11 (14.1%)
A small town	31 (39.7%)
A rural area	1 (1.3%)
	
***Health status perception***	
Very healthy	0 (0.0%)
Healthy	12 (15.2%)
Average	36 (45.6%)
Unhealthy	30 (38.0%)
Very unhealthy	1 (1.3%)
	
***Daily life perception***	
Very stressful	4 (5.1%)
Somewhat stressful	31 (39.2%)
Not too stressful	38 (48.1%)
Not stressful at all	6 (7.6%)

Furthermore, a huge proportion (80%) of the respondents was either privately- or self-employed and the majority (59%) lived in urban or semi-urban areas. When asked to rate their health status, nearly 40% of the TB patients who smoke rated themselves as 'unhealthy' or 'very unhealthy', while 45.6% believed that they were 'average'. The respondents were further asked about their perception of stress of daily life and nearly half (48.1%) perceived daily life as not too stressful.

The majority of the participants (78.5%) reported that they were in support of the on-going Malaysian government's campaigns against tobacco use (e.g.,"*Tak Nak*" or "Don't Want" program), whereas a marginal proportion (3.8%) was not. In addition, nearly one third (32.9%) of the smoking TB patients believed that the tobacco industry was truthful to the Malaysian people on the health dangers of tobacco use, whereas an equal percentage of the respondents had a contrary opinion.

Thirteen (16.3%) and 22 (27.5%) of the respondents reported that they started smoking at the age of 13 years or younger and 14-15 years, respectively. The study found that only 18.8% of the participants picked up the habit of smoking at an older age (20 years or older). When the nicotine dependence of the patients was measured using Fagerström Test for Nicotine Dependence (FTND) [22], more than half (51.3%) were moderately dependent (score of 4 - 6) and more than one-fourth (27.5%) were highly dependent on nicotine (score of 7 - 10). Furthermore, a moderately large proportion of the respondents (41.2%) reported that they have ever attempted to quit smoking, while more than half (56.3%) have not. On the other hand, a marginal proportion of 2.5% had previously been abstinent for more than six months. We further assessed the current stage of behavior change of the respondents using the transtheoretical model of change [23]. Half (50%) of them were in the preparation stage (willing and ready to quit within the next 30 days), while the remaining half were in either contemplation stage (considering quitting within the next 6 months but not in the next 30 days) or precontemplation stage (not thinking about quitting within the next 6 months). Additional details on the smoking-related characteristics of the study participants are presented in Table 2.

**Table 2:** Smoking-related characteristics of the newly diagnosed TB patients who smoke (N = 80)

Characteristic	n (%)
	
***Support the on-going government campaigns against tobacco use - e.g., "Tak Nak" program***	
Yes	62 (78.5%)
No	3 (3.8%)
Not sure	14 (17.7%)
	
***Truthfulness of tobacco industry to Malaysian people with regards to the serious health consequences of tobacco use***	
Yes	26 (32.9%)
No	26 (32.9%)
Not sure	27 (34.2%)
	
***Age of first time cigarette use***	
≤13 years	13 (16.3%)
14-15 years	22 (27.5%)
16-17 years	11 (13.8%)
18-19 years	19 (23.8%)
≥20 years	15 (18.8%)
	
***Fagerström Test for Nicotine Dependence (FTND)***	
High (7-10)	22 (27.5%)
Moderate (4-6)	41 (51.3%)
Minimal (<4)	17 (21.3%)
	
***Previous quitting smoking attempt***	
Have ever tried quitting smoking	33 (41.2%)
Have been abstinent for more than 6 months	2 (2.5%)
Have not tried quitting smoking before	45 (56.3%)
	
***Current stage of behavior change***	
Preparation	40 (50.0%)
Pre-contemplation or contemplation	40 (50.0%)

#### Tobacco Use Knowledge Scores among TB Patients who Smoke

Table 3 shows the proportions of the respondents who answered each item of tobacco use knowledge correctly. Less than half (47.5%) of the study participants had knowledge about the body system on which cigarette smoking has the greatest negative effect and slightly more than half (52.5%) understood the reason why smoker's heart works harder than that of non-smoker. In addition, only 35% correctly recognized that smokers get tired easily due to inability of their lungs to exchange gases effectively. Also, the majority wrongly believed that smokeless tobacco (snuff) can increase athletic performance (60%) and that it is a safe and harmless product (46.2%).

**Table 3:** Items measuring tobacco use knowledge among TB patients who smoke (N = 80)^a^

Knowledge Item	n (%) correct responses
Smoking has the greatest negative effect on the vascular system.	38 (47.5%)
A smoker's heart works harder because carbon monoxide makes the blood carry less oxygen.	42 (52.5%)
Nicotine, an ingredient in cigarette smoke, is both stimulating and depressing to the nervous system.	11 (13.8%)
Cigarette smokers get tired easily because their lungs cannot exchange gas well.	28 (35.0%)
The person most likely to get lung cancer is pipe smoker.	3 (3.8%)
Cigarette smokers are more likely to not live as long as non-smokers.	48 (60.0%)
The "smoker's cough", a type of chronic bronchitis is caused by irritation of the lungs and bronchial tubes and due to the chemicals in the cigarette.	54 (67.5%)
The dangers from cigarette smoking increase with dose (number of cigarettes smoked, number of years a person smoked, and amount of smoke inhaled).	20 (25.0%)
Smokeless tobacco is a safe, harmless product.	43 (53.8%)
Using chewing tobacco can lead to oral cancer.	48 (60.0%)
Using smokeless tobacco can increase athletic performance.	32 (40.0%)

^a^Eleven tobacco use knowledge items (total score of the participants ranged from 0 - 8)

Furthermore, only one-fourth of the respondents were able to recognize the dose-response relationship between smoking and related diseases. However, at least 60% of the participants knew that smokers are less likely to live as long as non-smokers and that chronic bronchitis in smokers is caused by irritation of the respiratory system and the chemicals in cigarettes (Table 3).

The mean (± SD) total score of tobacco use knowledge items was 4.23 ± 2.66. The influence of demographic and smoking-related characteristics on tobacco use knowledge was further tested using inferential statistics. Overall, the knowledge differed significantly with previous quit attempt, stage of change and ethnic groups (Table 4). Patients who were in the stage of contemplation/pre-contemplation had significantly less knowledge than those in the preparation stage of change (3.73 vs. 5.38; *p*=0.004). A similar trend was observed among those who had been abstinent for more than six months when compared with those who had never attempted quitting smoking.

**Table 4:** The influence of patient's characteristics on tobacco use knowledge

Characteristic	Mean ± SD of knowledge	*p*-value^a^
		
***Overall mean***	4.23 ± 2.66 (Range 0 - 8)	
		
***Age***		
18-25 years	5.00 ± 2.40	
26-33 years	3.00 ± 2.37	
34-41 years	5.67 ± 2.29	0.079
42-49 years	5.43 ± 2.47	
≥50 years	4.31 ± 2.63	
		
***Race***		
Malay	5.11 ± 2.38	
Chinese	2.94 ± 2.49	0.016
Indian	4.50 ± 2.52	
Others	3.50 ± 4.95	
		
***Occupation***		
Government	6.00 ± 1.63	
Private	4.51 ± 2.49	0.289
Self-employed	4.03 ± 2.85	
Others	5.42 ± 2.23	
		
***Living environment***		
An urban area	4.00 ± 2.93	
A suburban area	4.91 ± 2.47	
A small town	5.13 ± 2.06	0.299
A rural area	6.00	
		
***Marital status***		
Married	4.90 ± 2.66	
Single	4.19 ± 2.30	0.355
Divorced	3.50 ± 3.33	
Others	7.00	
		
***Health status perception***		
Very healthy	0	
Healthy	2.92 ± 3.09	
Average	5.08 ± 2.62	0.095
Unhealthy	4.57 ± 2.18	
Very unhealthy	4.00	
		
***Daily life perception***		
Very stressful	4.25 ± 3.10	
Somewhat stressful	4.97 ± 2.14	0.061
Not too stressful	3.89 ± 2.83	
Not stressful at all	6.67 ± 1.75	
		
***Support the on-going government campaigns against tobacco use***		
Yes	4.63 ± 2.65	
No	3.00 ± 1.00	0.574
Not sure	4.50 ± 2.59	
		
***Truthfulness of tobacco industry to Malaysian people with regards to the health consequences of tobacco***		
Yes	4.27 ± 2.78	
No	5.42 ± 2.34	0.098
Not sure	3.96 ± 2.52	
		
***Age of first time cigarette use***		
≤13 years	3.85 ± 2.48	
14-15 years	5.32 ± 2.26	
16-17 years	3.55 ± 2.81	0.328
18-19 years	4.58 ± 2.61	
≥20 years	4.73 ± 2.84	
		
***Fagerström Test for Nicotine Dependence (FTND)***		
High (7-10)	4.55 ± 2.50	
Moderate (4-6)	4.10 ± 2.60	0.114
Minimal (<4)	5.65 ± 2.45	
		
***Previous quitting smoking attempt***		
Have ever tried quitting	4.18 ± 2.71	
Have been abstinent for more than 6 months	7.50 ± 2.12	0.018
Have not tried quitting before	4.69 ± 2.45	
		
***Stage of change***		
Preparation	5.38 ± 2.12	0.004^b^
Pre-contemplation or contemplation	3.73 ± 2.76	

^a^One way ANOVA was applied; ^b^Independent *t*-test was used

#### Tobacco Use Attitudes of TB Smokers

The tobacco use attitudes of the study population were evaluated using an 18-item scale (Table 5). Notably, about two-thirds believed that smoking is fun (65.1%) and a similar proportion believed that it calms nerves (61.3%). Many respondents (70.1%) also agreed or strongly agreed that smoking makes them relieve all life stresses. However, an overwhelming proportion of the patients agreed or strongly agreed that: smoking is a waste of money (87.5%); tobacco use is very dangerous to health (91.3%) and; smokers are more likely to die from heart disease when compared with non-smokers (81.3%).

**Table 5:** Attitudes of newly diagnosed TB patients towards tobacco use (N = 80)

	Degree of Response				
	
Attitude Item	Strongly Disagree, n (%)	Disagree, n (%)	Neutral, n (%)	Agree, n (%)	Strongly Agree, n (%)
					
Smoking is fun.	3 (3.8%)	14 (17.5%)	11 (13.8%)	45 (56.3%)	7 (8.8%)
People smoke just to show off.	4 (5.0%)	23 (28.8%)	17 (21.3%)	30 (37.5%)	6 (7.5%)
Smoking calms your nerves.	2 (2.5%)	16 (20.0%)	13 (16.3%)	44 (55.0%)	5 (6.3%)
Smoking makes you smelly.	2 (2.5%)	6 (7.5%)	9 (11.3%)	49 (61.3%)	14 (17.5%)
Smoking makes you look tough.	3 (3.8%)	23 (28.8%)	25 (31.3%)	23 (28.8%)	6 (7.5%)
Smoking is a waste of money.	1 (1.3%)	3 (3.8%)	6 (7.5%)	34 (42.5%)	36 (45.0%)
Smoking makes you relieve all life stresses.	2 (2.5%)	6 (7.5%)	16 (20.0%)	49 (61.3%)	7 (8.8%)
Smoking keeps your weight down.	3 (3.8%)	7 (8.8%)	33 (41.3%)	32 (40.0%)	5 (6.3%)
Smoking gives you confidence.	3 (3.8%)	14 (17.5%)	36 (45.0%)	23 (28.8%)	4 (5.0%)
Smoking should be allowed at fewer places than it is now.	0 (0%)	10 (12.5%)	19 (23.8%)	41 (51.3%)	10 (12.5%)
Smoking is very dangerous to your health.	0 (0%)	1 (1.3%)	6 (7.5%)	35 (43.8%)	38 (47.5%)
Sales of cigarettes should be outlawed.	0 (0%)	1 (1.3%)	6 (7.5%)	47 (58.8%)	26 (32.5%)
People under 18 buying cigarettes should be restricted by law.	0 (0%)	3 (3.8%)	1 (1.3%)	35 (43.8%)	41 (51.3%)
Smoking gives you bad breath.	0 (0%)	3 (3.8%)	3 (3.8%)	43 (53.8%)	31 (38.8%)
Smokers are more likely to die from heart disease than non-smokers.	0 (0%)	3 (3.8%)	12 (15.0%)	41 (51.3%)	24 (30.0%)
It is okay to smoke if you don't get in the habit.	0 (0%)	11 (13.8%)	21 (26.3%)	40 (50.0%)	8 (10.0%)
Sharing cigarettes can act as an "ice breaker".	2 (2.5%)	14 (17.5%)	19 (23.8%)	35 (43.8%)	10 (12.5%)
Smoking together may lead to friendship.	3 (3.8%)	15 (18.8%)	21 (26.3%)	30 (37.5%)	11 (13.8%)

In addition, the vast majority had a positive attitude that: sales of cigarettes should be outlawed (91.3%), people below the age of 18 years should be restricted from purchasing cigarettes (95.1%), and smoking should be allowed at fewer places than it were (63.8%). Conversely, many respondents were neutral on the point that smoking keeps ones weight down (41.3%) and the belief that it gives confidence (45.0%).

#### Tobacco Use Behaviors among the Respondents

The pattern of tobacco products used among the participants is shown in Table 6. All the respondents had ever smoked cigarettes and admitted to annual cigarette smoking. Only one of them denied monthly cigarette smoking. The use of snuff was moderately prevalent among the participants with 23 respondents (28.8%) reporting ever snuffed. Of this, 17 (73.9%) and 14 (60.9%) reported annual and monthly snuffing, respectively. However, the use of cigar and pipe was uncommon among the study population (Table 6).

**Table 6:** Practice and pattern of tobacco products use among TB patients (N = 80)

Tobacco product use	n (%)
Ever smoked cigarettes	80 (100%)
Annual cigarettes smoking	80 (100%)
Monthly cigarettes smoking.	79 (98.8%)
Ever snuffed	23 (28.8%)
Annual snuffing	17 (21.3%)
Monthly snuffing	14 (17.6%)
Ever smoked cigars (tobacco)	8 (10.0%)
Annual cigar uses/smoking	5 (6.3%)
Monthly cigar uses/smoking	3 (3.8%)
Ever smoked a pipe (tobacco)	3 (3.8%)
Annual pipe uses	3 (3.8%)
Monthly pipe uses	2 (2.5%)

The study found that a higher proportion of the respondents started smoking cigarettes at younger age when compared with smokeless tobacco use (Table 7). The same patter holds true for other tobacco products. For instance, 7 of 8 (87.5%) cigar users and all 3 users of pipe (100%) started the behavior at the age of 20 years or older. The smoking initiation age for various tobacco products among the participants is presented in Table 7.

**Table 7:** Age of first-time use of various tobacco products among TB smokers (N = 80)

Age of first time use	n (%)
***Cigarette use***	
≤13 years	13 (16.3%)
14 - 15 years	22 (27.5%)
16 - 17 years	11 (13.8%)
18 - 19 years	19 (23.8%)
≥20 years	15 (18.8%)
	
***Snuff or smokeless tobacco use***	
≤13 years	0 (0%)
14 - 15 years	1 (1.3%)
16 - 17 years	3 (3.8%)
18 - 19 years	5 (6.3%)
≥20 years	13 (16.3%)
	
***Cigar use***	
≤13 years	0 (0%)
14 - 15 years	0 (0%)
16 - 17 years	1 (1.3%)
18 - 19 years	0 (0%)
≥20 years	7 (8.8%)
	
***Tobacco pipe use***	
≤13 years	0 (0%)
14 - 15 years	0 (0%)
16 - 17 years	0 (0%)
18 - 19 years	0 (0%)
≥20 years	3 (3.8%)

## Discussion

In addition to the collision between TB and tobacco epidemics in many developing nations, the prevalence of smoking among patients with TB is generally high [17,19]. Previous studies suggest that a large proportion of patients with TB may be active smokers or involuntarily exposed to other people's tobacco smoke [12,15-18,24]. In the present study, we found that smoking prevalence rate was high among patients with TB in the State of Penang (current and ex-smoking rates of 40.27% and 13.95%, respectively). This rate is as high as those reported from other countries (35 - 86%) [12,15,18,24-26]. Although the smoking rate in the current study is higher than the national average among the general adult population (21.5%), but it was somewhat lower than the male smoking rate in Malaysia (46.4%) [27]. Our findings may largely reflect smoking rate among male TB patients, since they predominated the study population. However, the rates in our study might have been grossly underestimated due to the unknown smoking status of a reasonable proportion of the newly diagnosed TB patients who might as well be tobacco smokers. Furthermore, since we used self-reported smoking status, it is possible that the rates might have been under-recorded, which would mean that the prevalence of smoking among patients with TB would be even greater than observed. A systematic review has demonstrated a trend of underestimation when smoking prevalence is based on self-reports [28]. One study from three West Africa countries (Guinea, Guinea Bissau, and The Gambia) reported that the smoking prevalence rate among TB cases was twice as high as among control household members (35% versus 17%, respectively) [15]. In India, the prevalence of smoking was 3.5 times as high among patients with TB compared with controls (86% versus 24%) [12].

Therefore, efforts should be geared towards reducing tobacco use among this population due to its negative impact on TB treatment outcomes. In the light of the burden of TB association with tobacco smoking, patients need to be well-educated about tobacco use and its health dangers. They also need to have positive attitudes against tobacco smoking. Malaysian government has initiated mass media campaigns against tobacco use under the "*Tak Nak*" or "Don't Want" program. Yet substantial proportions of TB patients in the current study were either not sure or did not support the on-going government campaigns against tobacco use.

Understanding the tobacco use knowledge, attitudes and behaviors of TB patients is of significance in the provision of behavioral therapy for smoking cessation. In a cross-sectional study among former TB patients in Indonesia, more than 30% of them reported that they were never asked about their smoking behavior or advised about quitting [26]. Such information will also be of value in designing effective educational intervention programs on motivating tobacco users to quit and urging non-users to avoid smoking. The educational programs can have an impact in the control and prevention of TB, treatment failure, relapse and poor prognosis. The mean total score of tobacco use knowledge of 4.23 (equivalent to 38.5%) found in this study suggests that newly diagnosed TB patients had poor knowledge of tobacco use. Although, the knowledge tested in the current study was about tobacco use and its health consequences in general, this finding points to possible deficits in knowledge specific to the association between tobacco smoke exposure and TB. Two recent studies conducted among ex-TB patients reported that the majority received only general health messages and not TB-specific messages [24,26]. Such subjects seem to be ill-informed about the impact of continued smoking on TB. Therefore educational programs specific to the impact of tobacco smoke exposure on TB should be designed to educate TB patients who are smokers on the general health dangers of tobacco use as well as its negative impacts on TB. They should also be enlightened on the short- and long-term benefits of quitting smoking on TB treatment outcomes and future lung health. In the present study, patients in the stage of preparation for behavior change were significantly more knowledgeable than their counterparts who were still in the contemplation and pre-contemplation stages (5.38 vs. 3.73, respectively). Perhaps this reaffirms that their intention to quit may be associated with the knowledge they possessed. Those who had previous quitting experience also seemed to be more knowledgeable than their counterparts who had never attempted to do so.

In general the patients had positive attitudes against tobacco use. These findings are encouraging. For instance, the respondents generally believed that tobacco smoking is a waste of money and is very dangerous to health. They also admitted that the sales of cigarettes should be outlawed, people under 18 years of age should be restricted from buying cigarettes and that smoking should be allowed at fewer places than it were. In this study, the use of cigar, pipe, and smokeless tobacco (snuff) was not prevalent among TB patients who smoke cigarettes. Furthermore, most of the respondents started the behavior of using tobacco products other than cigarettes at the age of 20 years and above. This indicates that intervention programs on prevention of tobacco use should target younger age groups.

This study has a number of limitations. First, the tobacco use behaviors of patients with TB may be under- or over-estimated in the light of using self-reports without biochemical verifications. Secondly, the sample may not be representative of all TB patients who are smokers in Malaysia, since it was derived from only two states. Lastly, the evaluation of knowledge did not include items specific to the negative impact of tobacco smoking on TB.

## Conclusion

Smoking prevalence among patients with TB in Malaysia was as high as those reported from other countries of the world. These patients generally had deficiencies in knowledge of tobacco use and its health dangers. In general, the study has managed to contribute additional information regarding the prevalence of smoking among newly diagnosed TB patients and their tobacco-use knowledge and attitudes. The results give cause for a great concern about the deficit in knowledge among TB patients who are smokers. Comprehensive tobacco education and smoking cessation programs should be aggressively promoted in TB settings.

## Competing interests

The authors declare that they have no competing interests.

## Authors' contributions

MHNM and AA conceived the research idea, designed the study and wrote its protocols. MHNM, AA, NMN, NAA, SASS, ARM, and AAM all contributed in the proposal writing. AA, MHNM, SASS, and NAA participated in the analysis and interpretation of the data. AA wrote the initial draft of the manuscript and MHNM edited it. NMN, NAA, SASS, ARM and AAM substantially helped in improving the intellectual contents and scientific merit of the entire manuscript.

## References

[B1] Pai M Mohan A Dheda K Leung CC Yew WW Christopher DJ Sharma SK Lethal interaction: the colliding epidemics of tobacco and tuberculosis Expert Rev Anti Infect Ther 2007 5 3 385 391 10.1586/14787210.5.3.385 17547503

[B2] WHO WHO report on the global tobacco epidemic 2008 Geneva, Switzerland: WHO 1 329

[B3] WHO WHO report on the global tuberculosis control 2008 Geneva, Switzerland WHO 1

[B4] Lin HH Ezzati M Murray M Tobacco smoke, indoor air pollution and tuberculosis: a systematic review and meta-analysis PLoS Med 2007 4 1 e20 10.1371/journal.pmed.0040020 17227135 PMC1769410

[B5] Bates MN Khalakdina A Pai M Chang L Lessa F Smith KR Risk of tuberculosis from exposure to tobacco smoke: a systematic review and meta-analysis Arch Intern Med 2007 167 4 335 342 10.1001/archinte.167.4.335 17325294

[B6] Slama K Chiang CY Enarson DA Hassmiller K Fanning A Gupta P Ray C Tobacco and tuberculosis: a qualitative systematic review and meta-analysis Int J Tuberc Lung Dis 2007 11 10 1049 1061 17945060

[B7] Davies PD Yew WW Ganguly D Davidow AL Reichman LB Dheda K Rook GA Smoking and tuberculosis: the epidemiological association and immunopathogenesis Trans R Soc Trop Med Hyg 2006 100 4 291 298 10.1016/j.trstmh.2005.06.034 16325875

[B8] d'Arc Lyra Batista J de Fatima Pessoa Militao de Albuquerque M de Alencar Ximenes RA Rodrigues LC Smoking increases the risk of relapse after successful tuberculosis treatment Int J Epidemiol 2008 37 4 841 851 18556729 10.1093/ije/dyn113 PMC2483312

[B9] Santha T Garg R Frieden TR Chandrasekaran V Subramani R Gopi PG Selvakumar N Ganapathy S Charles N Rajamma J Risk factors associated with default, failure and death among tuberculosis patients treated in a DOTS programme in Tiruvallur District, South India, 2000 Int J Tuberc Lung Dis 2002 6 9 780 788 12234133

[B10] Thomas A Gopi PG Santha T Chandrasekaran V Subramani R Selvakumar N Eusuff SI Sadacharam K Narayanan PR Predictors of relapse among pulmonary tuberculosis patients treated in a DOTS programme in South India Int J Tuberc Lung Dis 2005 9 5 556 561 15875929

[B11] Chang KC Leung CC Tam CM Risk factors for defaulting from anti-tuberculosis treatment under directly observed treatment in Hong Kong Int J Tuberc Lung Dis 2004 8 12 1492 1498 15636497

[B12] Gajalakshmi V Peto R Kanaka TS Jha P Smoking and mortality from tuberculosis and other diseases in India: retrospective study of 43000 adult male deaths and 35000 controls Lancet 2003 362 9383 507 515 10.1016/S0140-6736(03)14109-8 12932381

[B13] WHO Tobacco or health: A global status report 1997 Geneva: WHO

[B14] Zellweger JP Tobacco and tuberculosis Monaldi Arch Chest Dis 2008 69 2 83 85 18837424 10.4081/monaldi.2008.403

[B15] Lienhardt C Fielding K Sillah JS Bah B Gustafson P Warndorff D Palayew M Lisse I Donkor S Diallo S Investigation of the risk factors for tuberculosis: a case-control study in three countries in West Africa Int J Epidemiol 2005 34 4 914 923 10.1093/ije/dyi100 15914505

[B16] Schneider NK Novotny TE Addressing smoking cessation in tuberculosis control Bull World Health Organ 2007 85 10 820 821 10.2471/BLT.07.034797 18038065 PMC2636489

[B17] Yach D Partnering for better lung health: improving tobacco and tuberculosis control Int J Tuberc Lung Dis 2000 4 8 693 697 10949319

[B18] Wang J Shen H Review of cigarette smoking and tuberculosis in China: intervention is needed for smoking cessation among tuberculosis patients BMC Public Health 2009 9 292 10.1186/1471-2458-9-292 19674472 PMC2734854

[B19] Siddiqi K Lee AC An integrated approach to treat tobacco addiction in countries with high tuberculosis incidence Trop Med Int Health 2009 14 4 420 428 10.1111/j.1365-3156.2009.02238.x 19222822

[B20] Torabi MR Yang J Li J Comparison of tobacco use knowledge, attitude and practice among college students in China and the United States Health Promot Int 2002 17 3 247 253 10.1093/heapro/17.3.247 12147639

[B21] Meier KS Tobacco truths: the impact of role models on children's attitudes toward smoking Health Educ Q 1991 18 2 173 182 2055775 10.1177/109019819101800203

[B22] Heatherton TF Kozlowski LT Frecker RC Fagerstrom KO The Fagerstrom Test for Nicotine Dependence: a revision of the Fagerstrom Tolerance Questionnaire Br J Addict 1991 86 9 1119 1127 10.1111/j.1360-0443.1991.tb01879.x 1932883

[B23] DiClemente CC Prochaska JO Fairhurst SK Velicer WF Velasquez MM Rossi JS The process of smoking cessation: an analysis of precontemplation, contemplation, and preparation stages of change J Consult Clin Psychol 1991 59 2 295 304 10.1037/0022-006X.59.2.295 2030191

[B24] Pradeepkumar AS Thankappan KR Nichter M Smoking among tuberculosis patients in Kerala, India: proactive cessation efforts are urgently needed Int J Tuberc Lung Dis 2008 12 10 1139 1145 18812043

[B25] Altet-Gomez MN Alcaide J Godoy P Romero MA Hernandez del Rey I Clinical and epidemiological aspects of smoking and tuberculosis: a study of 13,038 cases Int J Tuberc Lung Dis 2005 9 4 430 436 15830749

[B26] Ng N Padmawati RS Prabandari YS Nichter M Smoking behavior among former tuberculosis patients in Indonesia: intervention is needed Int J Tuberc Lung Dis 2008 12 5 567 572 18419894

[B27] Ministry of Health Malaysia Report of the Third National Health and Morbidity Survey 2006 Public Health Institute, Minstry of Health Malaysia

[B28] Gorber SC Schofield-Hurwitz S Hardt J Levasseur G Tremblay M The accuracy of self-reported smoking: a systematic review of the relationship between self-reported and cotinine-assessed smoking status Nicotine Tob Res 2009 11 1 12 24 10.1093/ntr/ntn010 19246437

